# Neuropathic Corneal Pain and Blepharospasm: A Case Series

**DOI:** 10.3390/diagnostics16131974

**Published:** 2026-06-25

**Authors:** Zhang Zhe Thia, Aya Takahashi, Mingyi Yu, Chang Liu, Isabelle Xin Yu Lee, Louis Tong, Yu-Chi Liu

**Affiliations:** 1 Regenerative Therapy Group, Singapore Eye Research Institute, Singapore 169856, Singapore; thiazz1995@hotmail.com (Z.Z.T.); aya.nakajima@snec.com.sg (A.T.); yu.mingyi@seri.com.sg (M.Y.); chang.liu@seri.com.sg (C.L.); isabelle.lee.x.y@seri.com.sg (I.X.Y.L.); 2 Cornea & External Eye Disease, Singapore National Eye Centre, Singapore 169856, Singapore; louis.tong.h.t@singhealth.com.sg; 3 Ocular Surface Research Group, Singapore Eye Research Institute, Singapore 169856, Singapore; 4 Ophthalmology and Visual Sciences Academic Clinical Program, Duke-NUS Medical School, Singapore 169857, Singapore

**Keywords:** neuropathic corneal pain, blepharospasm

## Abstract

***Background and Clinical Significanc:*** Neuropathic corneal pain is a debilitating condition characterized by ocular pain disproportionate to clinical signs, often resulting from peripheral and central sensitization of the corneal somatosensory pathway. Emerging evidence suggests that chronic involuntary muscle contraction in blepharospasm may lead to irritation of trigeminal afferents and corneal neurogenic inflammation, potentially predisposing patients to neuropathic corneal pain. Given its debilitating nature, early recognition can prevent the progression of neuropathic sequelae. This study examines the potential role of blepharospasm as a predisposing factor contributing to neuropathic corneal pain. ***Case Presentation:*** This retrospective case series describes three cases (median age: 50 years) of neuropathic corneal pain in association with blepharospasm and their clinical course following multimodal treatment over a median follow-up period of one year. Ocular surface was evaluated using slit-lamp biomicroscopy, while corneal nerve structure and morphology were assessed with in vivo confocal microscopy. All the three subjects presented with minimal ocular surface staining but disproportionate ocular pain characterized by burning sensation and photophobia. Proparacaine challenge testing was performed to determine the subtype of neuropathic corneal pain. Pain symptoms and quality of life were evaluated using the Ocular Pain Assessment Survey and Ocular Surface Disease Index questionnaires. In vivo confocal microscopy demonstrated characteristic corneal nerve abnormalities including reduced corneal nerve density, increased nerve tortuosity, and the presence of microneuromas. Treatment included oral Pregabalin or Gabapentin, topical lubricants, Cyclosporine 0.05% (1 case), and 20% autologous serum eye drops (1 case). Two of the three cases received four to five injections of botulinum toxin for blepharospasm, whereas one had undergone a single injection prior to review. All patients also received weekly periorbital quantum molecular resonance electrotherapy for two months. Improvements were observed across multiple domains of the Ocular Pain Assessment Survey and Ocular Surface Disease Index evaluation, including ocular pain, photophobia, non-ocular pain, and quality-of-life measures following multimodal treatment. The co-existence of blepharospasm and neuropathic corneal pain observed in our cases supports a possible association between chronic periocular muscle hyperactivity and corneal nociceptor sensitization. Proposed mechanisms include chronic trigeminal nerve irritation, neurogenic inflammation, and sensitization mediated by pro-inflammatory neuropeptides. Multimodal treatment targeting both motor hyperactivity and neuropathic pain pathways appeared to provide symptomatic relief, including the use of quantum molecular resonance electrotherapy, which might modulate pain pathways, block nociceptor neurotransmission, and accelerate corneal nerve regeneration. Given the complexity of the neural pathways responsible for ocular discomfort, further studies are required to elucidate the relationship between neuropathic corneal pain and blepharospasm in larger cohorts, as well as refine existing therapeutic approaches, including evaluating the therapeutic role of electrotherapy. ***Conclusions:*** Blepharospasm may represent a potential predisposing factor of neuropathic corneal pain. Early recognition and concurrent treatment of blepharospasm and neuropathic corneal pain can effectively relieve symptoms and improve quality of life. Adopting a multimodal treatment approach is therefore recommended.

## 1. Introduction

Ocular pain can be classified into either nociceptive or neuropathic pain [1]. Neuropathic corneal pain (NCP) arises from a lesion within the somatosensory system and involves peripheral axonal injuries, leading to the release of pro-inflammatory neuropeptides [2]. The pathophysiology of NCP results in peripheral sensitization [3], which, in turn, causes central sensitization and heightened pain awareness over time [2,4]. Symptoms such as hyperalgesia, allodynia, photoallodynia, spontaneous pain, itching, irritation, aching, dryness, foreign body sensation, pressure, or burning sensation, are often out of proportion to clinical signs [5,6], with only minimal findings on ocular surface staining observed via slit-lamp examination [7]. Diagnosis remains challenging because there is no standardized diagnostic criteria, and evaluation currently relies on clinical history, symptom assessment, proparacaine challenge testing, and imaging tests such as in vivo confocal microscopy (IVCM) findings [6]. IVCM can be helpful in diagnosing NCP by demonstrating characteristic corneal nerve abnormalities, particularly corneal microneuromas, which have been increasingly recognized as a potential imaging biomarker of NCP [8]. Other reported findings include reduced corneal nerve fiber density (CNFD), increased nerve tortuosity or beading, activated stromal keratocytes, and a honeycomb-like appearance in the stroma [2,9]. Clinically, the proparacaine challenge test has been used as a functional assessment tool to differentiate between the peripheral, central, or mixed subtypes of NCP. Complete symptom relief following topical proparacaine instillation suggests predominantly peripheral sensitization, partial relief indicates mixed peripheral and central sensitization, while persistent pain is suggestive of central sensitization [6,10]. Nevertheless, NCP remains a poorly defined entity, and the variability in symptoms, lack of clinical signs, and significant overlap with conditions such as dry eye disease (DED) make diagnosis challenging, with NCP currently being largely a diagnosis of exclusion [3,6].

NCP can negatively impact overall quality of life and imposes a significant psychological and economic burden [11,12]. Validated pain questionnaires enable clinicians to evaluate patients’ symptoms and the changes in their quality of life. The Ocular Pain Assessment Survey (OPAS) is a quantitative, multi-dimensional questionnaire specifically designed for the assessment of ocular pain. Questions are grouped into six distinct dimensions that include eye pain intensity in the past 24 h, eye pain intensity in the past 2 weeks, non-eye pain questions, quality of life, aggravating factors, and associated symptoms [13]. Patients were asked to respond to all questions according to the pain scale or frequency between 0 and 10, or 0% to 100% (0 = no pain, 10 or 100% = worst pain ever) [14]. Similarly, the Ocular Surface Disease Index (OSDI) is a reliable tool consisting of 12 questions that explore symptoms and their impact on vision-related function. Both questionnaires serve to monitor and compare ocular pain symptoms, allowing for better understanding of the impact of NCP on patients’ quality of life [12]. Sub-optimally managed symptoms can disrupt crucial daily activities, giving the subject a sense of handicap [2]. Severe cases may even experience physical, psychological, and functional impairment, with significant reduction in quality of life as a result of mood disorders, sleep disruptions, and poorer social relationships [12].

Etiologies of NCP can be classified into either ocular or systemic causes. The former includes corneal surgery such as refractive procedures, ocular trauma, DED, recurrent corneal erosion syndrome, infectious or herpetic keratitis, long-term contact lens wear, chronic ocular surface inflammation, chemical injury, and ultraviolet exposure. The latter includes diabetes mellitus, trigeminal neuralgia, fibromyalgia, migraine, autoimmune conditions, small fiber neuropathy, and medication-induced neuropathy such as chemotherapy [1,3,6,15]. Psychological comorbidities such as anxiety, depression, and post-traumatic stress disorders also predispose patients to NCP [3]. Given its debilitating nature, broad etiologic spectrum, and paucity of data, more research is needed to further our understanding of NCP and its potential risk factors.

One such association is blepharospasm [11,16]. While the available data confirming this relationship remain limited, emerging evidence appears to suggest a possible link between blepharospasm and the development of neuropathic-like pain symptoms. A study by Scorr et al. reported a high frequency of photophobia, ocular pain, and a gritty or burning sensation in patients with blepharospasm [17]. Among those features, burning sensation and photophobia have been reported to be the two most common symptoms in patients with NCP [2]. Furthermore, a number of studies documented that the use of Botox injection for motor suppression achieved significant improvement or resolution of photophobia [18], neuropathic ocular pain and facial pain [19,20,21,22]. Such response patterns indirectly support the link between sustained involuntary contraction contributing to neuropathic-like pain. In addition, the proposed neuromodulatory effects of peripheral electrotherapy, together with its reported benefits in recalcitrant DED [23], have generated interest in its potential application as a non-invasive adjunctive therapy for conditions involving ocular surface neurosensory dysfunction. Integrating electrotherapy into existing pain management strategies may enhance pain relief for patients with refractory NCP [24]. Here, we discuss three patients with blepharospasm presenting with NCP, examining their clinical course following multimodal treatment incorporating periorbital electrotherapy (Quantum Molecular Resonance (QMR), Rexon-Eye^®^, Sandrigo, Italy).

## 2. Case Presentation

These three patients were diagnosed with NCP based on the published inclusion criteria [2,25]: (1) persistent ocular pain or pain-like symptoms for at least three months, including burning sensation, allodynia, photoallodynia, or hyperalgesia, with a minimum 30% rating on more than three questions in the OPAS questionnaire; (2) corneal nerve abnormalities on IVCM evaluation such as microneuroma formation, beading pattern, nerve tortuosity, reduction in CNFD or corneal nerve fiber length (CNFL); (3) minimal or absence of ocular surface staining, with National Eye Institute (NEI) dot-count scores less than 2; and (4) no response to conventional dry eye treatments for at least six months. This study was conducted in Singapore National Eye Centre and Singapore Eye Research Institute. Approval for the study was granted by the Institutional Review Board of SingHealth (number 2022-2421), Singapore. The study was conducted in accordance with the Declaration of Helsinki, and informed consent was obtained from all the subjects. Pain symptoms were evaluated using the OPAS and OSDI questionnaires [12]. A summary of the clinical presentations, treatment modalities, and outcomes of all three cases is provided in Table 1.

**Figure 1:**
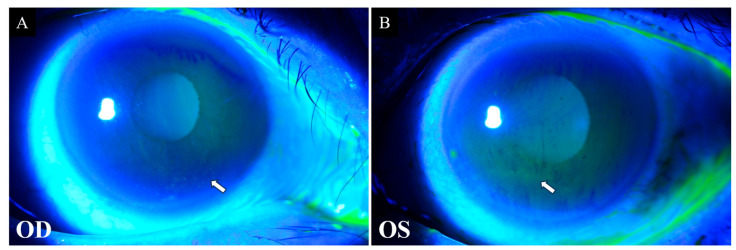
Slit-lamp biomicroscopy demonstrating minimal fluorescein staining at the inferior corneas of the right eye (OD) (**A**) and left eye (OS) (**B**); arrows. Original magnification ×16.

**Figure 2:**
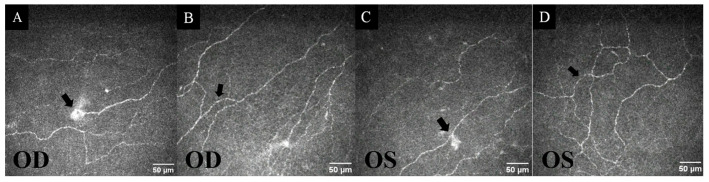
Representative IVCM images at the first presentation showing multiple neuromas (arrows) in both eyes, larger in the right eye (**A**,**B**) compared to the left eye (**C**,**D**), with increased nerve tortuosity. Scale bar: 50 µm.

Case 1 is a 50-year-old Chinese female with a history of DED who underwent uncomplicated bilateral blepharoplasty more than 20 years prior. She subsequently developed ocular pain characterized by a severe burning sensation (100% rating) and photophobia (50% rating), accompanied by periocular spasms, resulting in excessive involuntary blinking. Her symptoms remained inadequately controlled despite conventional dry eye therapies (e.g., lubricants) at a private clinic, with progressive deterioration over time significantly impacting her quality of life, mood, and mental health. Slit-lamp examination revealed minimal fluorescein staining of the inferior corneas (Figure 1A,B). Ocular surface assessment showed a Schirmer’s I test result to be 2 mm, Oxford and NEI grades of 2, and tear break-up times (TBUT) of 1 and 2 s in the right and left eyes, respectively. Corneal sensitivity, assessed using a Cochet–Bonnet aesthesiometer (Luneau Ophthalmologia, Pont-de-l’Arche, France) at each quadrant and central area of the cornea, had a total value of 30 cm, suggesting normal corneal sensitivity. IVCM demonstrated multiple corneal neuromas, increased nerve tortuosity and beading (Figure 2A–D) on both eyes. A proparacaine challenge test revealed no significant pain relief at five minutes, with pain scores of 8 and 7 pre-and post-instillation, respectively. The patient was diagnosed with central-type NCP.

The patient received 20 units of Botox injection, Pregabalin 200 mg three times daily, Gabapentin 600 mg three times daily, 20% autologous serum eye drops eight times daily, and 0.1% Fluorometholone eye drops twice daily. She also underwent weekly periorbital QMR electrotherapy sessions for two months, with each session lasting 30 min. Three weeks after the above-mentioned treatment, her total OSDI score improved from 61 to 27, with reductions in burning sensation and photophobia to 80% and 50%, respectively. OPAS results further demonstrated improvement in overall pain, with the worst non-ocular pain over the past 24 h and two weeks both decreasing from 60% to 0%. Post-treatment corneal sensitivity remained stable at 30 cm on Cochet–Bonnet aesthesiometry.

**Figure 3:**
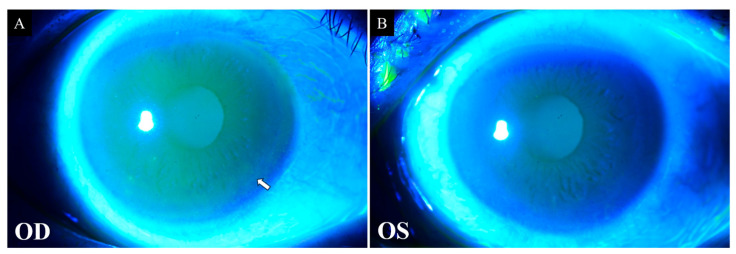
Slit-lamp biomicroscopy demonstrating minimal fluorescein staining at the inferior cornea of the right eye (OD) (**A**); arrow. No fluorescein staining of the cornea and conjunctiva of the left eye (OS) (**B**). Original magnification ×16.

**Figure 4:**
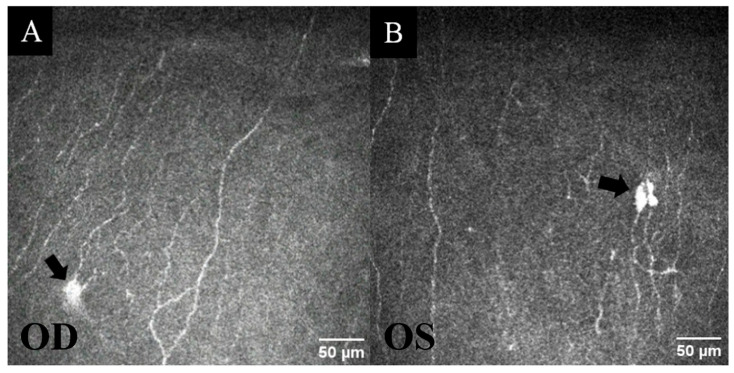
Representative IVCM images showing 1 to 2 small neuromas in both right (**A**); arrow and left (**B**) arrow eyes. Scale bar: 50 µm.

Case 2 is a 45-year-old Chinese male with a history of migraine, on long-term follow-up for meibomian gland dysfunction, who expressed concerns of photophobia (95% rating) and burning ocular discomfort (70% rating). Frequent eyelid twitching was observed during review, and a diagnosis of blepharospasm was made. Schirmer’s I test was 1 mm in both eyes, with Oxford and NEI staining grades of 1. Corneal sensitivity measured 30 cm across all corneal regions, and slit-lamp examination revealed minimal fluorescein staining of the right eye (Figure 3A,B). IVCM demonstrated a few small corneal neuromas in both eyes (Figure 4A,B). The patient was diagnosed with mixed-type NCP after the proparacaine challenge test, with VAS scores improving from 3 to 1 after instillation. Blepharospasm was effectively controlled following the injection of 20 units of Botox to both periorbital regions. Pregabalin 150 mg twice daily and 0.05% Cyclosporine eye drops twice daily were also initiated in addition to the usual lubricants, followed by weekly periorbital QMR electrotherapy sessions for two months. The patient subsequently reported notable improvement in photophobia (50% rating) and burning sensation (50% rating), with OSDI score improving from 63 to 33. OPAS results also demonstrated improvement in quality-of-life measures across different aspects of daily activities, decreasing from 90–95% to 50%.

Case 3 describes a 56-year-old Chinese male with a history of blepharospasm as part of Meige syndrome, who was referred to our NCP clinic for evaluation of ocular pain associated with photophobia (50% rating). Corneal sensitivity measured 20 cm and 30 cm for the right and left eye, respectively. He was diagnosed with mixed-type NCP following the proparacaine challenge test, which showed VAS scores of 2 before and 1 after instillation. Improvement in eyelid twitches was noted after receiving 68 units of Botox injection to both periorbital regions, with repeat injections every one to three months. Weekly periorbital QMR electrotherapy was also completed over two months, in addition to topical lubricants. Following these interventions, the patient experienced sustained symptomatic relief, with resolution of photophobia (0% rating) and improvement in OSDI scores from 31 to 18. OPAS results indicated resolution of overall pain from 70–80% to 0%, as well as the worst non-ocular pain in the past 24 h and two weeks, both reducing from 60% to 0%. Quality-of-life scores across domains such as general activity (60% rating), mood (100% rating), and enjoyment of life and interpersonal relationships (50% rating) all improved to 0%. Post-treatment corneal sensitivity improved from 20 cm to 30 cm in the right eye and remained stable at 30 cm in the left eye.

## 3. Discussion and Conclusions

Blepharospasm is a subtype of dystonia characterized by bilateral synchronous spasms of the orbicularis oculi and surrounding muscles [26,27]. This continuous muscular contraction associated with dystonic muscular disorders may induce a severe chronic pain syndrome [28]. Although the link between blepharospasm and NCP remains under-reported, studies found evidence of non-motor sensory discomfort, such as photophobia, pain, and ocular burning sensation being common in blepharospasm cohorts [17,29]. This may indirectly suggest the possibility of blepharospasm being accompanied by, or even predispose patients to, the development of neuropathic-type sensory symptoms.

While the mechanism leading to the development of NCP has yet to be conclusively proven, we hypothesize that NCP may be induced by the chronic irritation of trigeminal nerve fibers as a result of continuous contraction of the neighboring muscle fibers. Numerous descriptions in the literature on patients with chronic pain-associated dystonias achieving neuropathic ocular pain relief following Botox injections further support this relationship [30,31]. Therefore, symptom relief occurs through the direct attenuation of muscle contractions by cholinergic denervation at motor end-plates after Botox injections [32]. As ocular surface disturbances such as light can trigger the trigeminal nerve pathway through the activation of first-order neurons within the ophthalmic division, Botox use in decreasing facial muscle contraction may be beneficial in improving photophobia by acting at this level of the pathway and blocking pain signaling through the primary trigeminal afferent [18,33]. We believe that symptom relief can also be attributed to a reduction in activity involving areas of the brain that process pain, as evidenced on functional magnetic resonance imaging [18].

Besides irritation of trigeminal afferents, another potential mechanism involves neurogenic inflammation, where chronic involuntary muscle contraction leads to mechanical hyperalgesia and an increased release of neuropeptides from the peripheral terminals of corneal nociceptors, whose cell bodies reside in the trigeminal ganglion [34,35]. One such neuropeptide is calcitonin gene-related peptide (CGRP), which is released from the trigeminal neurons [33,36]. This can provoke neurogenic inflammation, sensitize trigeminal afferents, and increase pain perception by potentiating the effect of other nociceptive neuropeptides [36,37,38]. Another neuropeptide, substance P, is responsible for the activation of neurogenic inflammatory processes from the trigeminal nerve endings [22]. It directs immune cells to the terminals of nociceptors where neurokines are released, contributing to neuropathic pain [39]. These pro-inflammatory neuropeptides have prolonged effects on corneal nociceptors and are strongly implicated in sensory hypersensitivity, triggering photophobia [37]. Meanwhile, patients with NCP may also develop secondary hyperalgesia within the trigeminal nerve distribution, presenting with blepharospasm and generalized discomfort around the orbit and face [40], resulting in a vicious cycle.

Botulinum toxin can potentially normalize pain sensitivity by inhibiting the release of pro-inflammatory substrates from peripheral corneal nociceptors [36,41], through the inactivation of transport proteins including synaptosomal-associated protein 25 (SNAP-25) and vesicle-associated membrane protein (VAMP) [42]. A study by Reyes et al. further discussed the mechanisms in which Botox may act to reduce CGRP release, thereby reducing sensitization and leading to improved symptoms of pain and photophobia [18]. The two mechanisms proposed include decreasing facial muscle contraction and increasing the cleavage of SNAP-25 which blocks synaptic exocytosis [31,43].

Relief of blepharospasm and ocular pain after electrotherapy was observed in all our cases, consistent with emerging evidence supporting the role of peripheral electrotherapy in the management of NCP [24]. Post-treatment Cochet–Bonnet aesthesiometry, available in two of the cases examined, demonstrated preservation of corneal sensitivity following treatment, without evidence of treatment-related sensory impairment. These findings support the notion that peripheral electrotherapy may promote neuromodulation and corneal nerve recovery without impairing physiological corneal sensation. However, post-treatment measurements were unavailable in one patient, and larger studies incorporating serial corneal sensitivity assessments are required to better characterize the sensory effects of electrotherapy.

Studies have suggested that transcutaneous electrotherapy modulates pain pathways, blocks nociceptor neurotransmission [44], and accelerates corneal nerve regeneration [24,45]. For instance, pain modulation in NCP may be mediated by nerve-stimulating effects, including the induction of nerve growth factors expression, leading to accelerated corneal nerve regeneration via increased calcium influx into ganglion neurons and enhanced corneal sensitivity [45,46,47]. Among different electrotherapies, transcutaneous electrical nerve stimulation (TENS) is a widely used form that involves the placement of electrodes near the site of pain, stimulating the nerves and alleviating pain through low-frequency electrical impulses and the reduction in nociceptor activity. This modulates the expression of pain-related ion channels, blocking nociceptor neurotransmission [44,48]. A reduction in the release of neurotransmitters glutamate and substance P in the spinal cord further decreases excitability and the risk of central sensitization following high-frequency TENS [49]. It is also believed that the sympatholytic effect of TENS plays a role in pain modulation, since sympathetic efferent hyperactivity contributes to the development and persistence of NCP [50].

Among the available modalities of electrotherapy, QMR electrotherapy is a non-invasive treatment platform that delivers low-intensity, high-frequency electrical currents to periocular tissues through contact electrodes [51]. Previous studies have demonstrated that QMR electrotherapy improved tear film stability, ocular surface symptoms, and quality-of-life measures in patients with recalcitrant DED [23,51,52,53]. Its proposed therapeutic effects include the enhancement of cellular metabolism, tissue regeneration, microcirculation, and neuromodulation, which may potentially contribute to ocular surface recovery and pain modulation [23].

While emerging studies suggest peripheral electrotherapy as a promising non-invasive treatment option for NCP, its role in the management of blepharospasm remains incompletely understood. A recent randomized pilot study evaluating TENS for neuropathic or nociplastic ocular pain demonstrated improvement in ocular pain symptoms and highlighted the therapeutic role of peripheral neuromodulation in ocular surface pain disorders, with treatment response potentially associated with a reduced disruption of central pain modulatory mechanisms [54]. In addition, studies by Zeng et al. and Gittens et al. demonstrated the plausibility of electrotherapy in potentially modifying eyelid motor control and blink reflexes [55,56]. Such neuromodulatory effects may potentially contribute to the improvement in blepharospasm observed in our cases following QMR electrotherapy. Further research is required to evaluate the therapeutic efficacy of peripheral electrical stimulation in NCP and blepharospasm modulation, as well as determine the optimal parameters and protocol, including treatment duration, frequency, and intensity, before electrotherapy can be widely integrated into existing management strategies.

In summary, despite the increasing awareness of NCP in recent years, important gaps in our understanding of this condition remain. Given the complexity of the neural pathways responsible for ocular discomfort, future research should focus on elucidating the relationship between NCP and blepharospasm on a molecular level in larger cohorts, as well as refining existing therapeutic approaches. This case series highlights that blepharospasm could predispose individuals to NCP. Our findings underscore the importance of early recognition and targeted motor suppression to achieve symptom control in these patients and prevent the progression of neuropathic sequelae. Multimodal treatment is recommended for effective pain relief and blepharospasm control.

## Figures and Tables

**Table 1:** Summary of the clinical findings, treatment modalities, and outcomes of the three cases of blepharospasm-associated NCP.

Case	NCP Type	Clinical Presentations	Existing Treatment(s)	Additional Treatment(s) Introduced During Study Period	Outcomes
1	Central	Severe burning sensation (100%) Photophobia (50%) Periocular spasms Corneal staining minimal Corneal sensitivity normal Multiple corneal neuromas on IVCM	Lubricants	Botox 20 units Pregabalin 200 mg three times daily Gabapentin 600 mg three times daily 20% autologous serum tears eight times daily 0.1% Fluorometholone twice daily QMR electrotherapy weekly for 2 months	Burning sensation decreased to 80% OSDI improved: 61 → 27 Improvement in overall pain and resolution of non-ocular pain on OPAS Corneal sensitivity preserved after treatment
2	Mixed	Photophobia (95%) Burning sensation (70%) Frequent eyelid twitching Corneal staining minimal Corneal sensitivity normal Few corneal microneuromas on IVCM	Lubricants	Botox 20 units Pregabalin 150 mg twice daily Cyclosporine 0.05% twice daily QMR electrotherapy weekly for 2 months	Burning sensation improved to 50% Photophobia improved to 50% OSDI improved: 63 → 33 Improvement in quality-of-life measures on OPAS
3	Mixed	Ocular pain with photophobia (50%) Eyelid twitching Corneal sensitivity reduced in the right eye	Botox 68 units with repeat injections every 1–3 months Lubricants	QMR electrotherapy weekly for 2 months	Resolution of photophobia (0%) OSDI improved: 31 → 18 Resolution of overall pain and non-ocular pain on OPAS Improvement in quality-of-life measures on OPAS Corneal sensitivity improved after treatment

The outcomes reflect overall clinical improvement following multimodal treatment. In Case 3, QMR electrotherapy was introduced in addition to an established Botox regimen.

## Data Availability

All data generated or analyzed during this study are included in this published article.

## Abbreviations

NCP	Neuropathic corneal pain
IVCM	In vivo confocal microscopy
CNFD	Corneal nerve fiber density
CNFL	Corneal nerve fiber length
NEI	National Eye Institute
DED	Dry eye disease
QMR	Quantum Molecular Resonance
VAS	Visual Analogue Scale
OPAS	Ocular Pain Assessment Survey
OSDI	Ocular Surface Disease Index
CGRP	Calcitonin gene-related peptide
SNAP-25	Synaptosomal-associated protein 25
VAMP	Vesicle-associated membrane protein
TENS	Transcutaneous electrical nerve stimulation
